# Rewiring Carbon Metabolism in *Bacillus methanolicus* via Heterologous Phosphoketolase Expression Enhances Biomass Yield From Methanol and Reduces CO_2_ Loss

**DOI:** 10.1111/1751-7915.70430

**Published:** 2026-09-01

**Authors:** Yael J. Toporek, Jonathan R. Humphreys, Thomas J. Musselwhite, Marcus S. Bray, Katie P. Michel, Kimberly A. Rosenbach, Michael T. Guarnieri

**Affiliations:** ^1^ Bioenergy & Sustainable Transportation Directorate, National Laboratory of the Rockies Golden Colorado USA; ^2^ Department of Chemical and Biological Engineering Colorado School of Mines Golden Colorado USA; ^3^ Renewable & Sustainable Energy Institute University of Colorado Boulder Colorado USA

**Keywords:** *Bacillus methanolicus*, biomass yield, methylotrophy, phosphoketolase

## Abstract

Methylotrophic microbes are attractive alternatives to traditional heterotrophic production platforms, yet their efficiency is constrained by carbon loss through pyruvate decarboxylation and the oxidative branch of the RuMP cycle. The phosphoketolase (PKT) pathway provides a carbon‐conserving alternative by cleaving fructose‐6‐phosphate and/or xylulose‐5‐phosphate into acetyl‐phosphate, which can subsequently be converted to acetyl‐coA without pyruvate decarboxylation. The remaining carbon intermediates are recycled through central metabolism to regenerate RuMP cycle intermediates without direct CO_2_ release. Here, we engineered this strategy in *Bacillus methanolicus* , a thermophilic methylotroph with strong industrial potential. We first established a versatile expression toolkit comprising inducible and constitutive promoters, benchmarked using an sfGFP reporter. Leveraging this system, we heterologously expressed the phosphoketolase B (*pktB*) gene from *Methylotuvimicrobium buryatense* 5GB1C which increased methanol‐to‐biomass yields by 18%–24% relative to controls and reduced biogenic CO_2_ production by 9%–12%. Chromosomal integration of *pktB* preserved these gains, demonstrating stability without reliance on plasmid‐based expression. Together, these results show that PKT‐driven metabolic rewiring enhances substrate yields in *B. methanolicus* and provides a scalable strategy to improve methylotrophic bioprocesses. This work expands the metabolic engineering toolbox for methylotrophs and highlights carbon‐conserving pathway design as a key lever for advancing single carbon (C1) biomanufacturing.

## Graphical abstract

Introducing a phosphoketolase pathway into *Bacillus methanolicus* enhances methanol‐to‐biomass yields by 18%–24% and reduces CO_2_ evolution by up to 12%. Coupled with a tunable expression system, this work demonstrates improved carbon conservation and expands metabolic engineering strategies for methylotrophs.

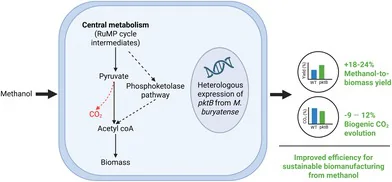

## 1 Introduction

**FIGURE 1:**
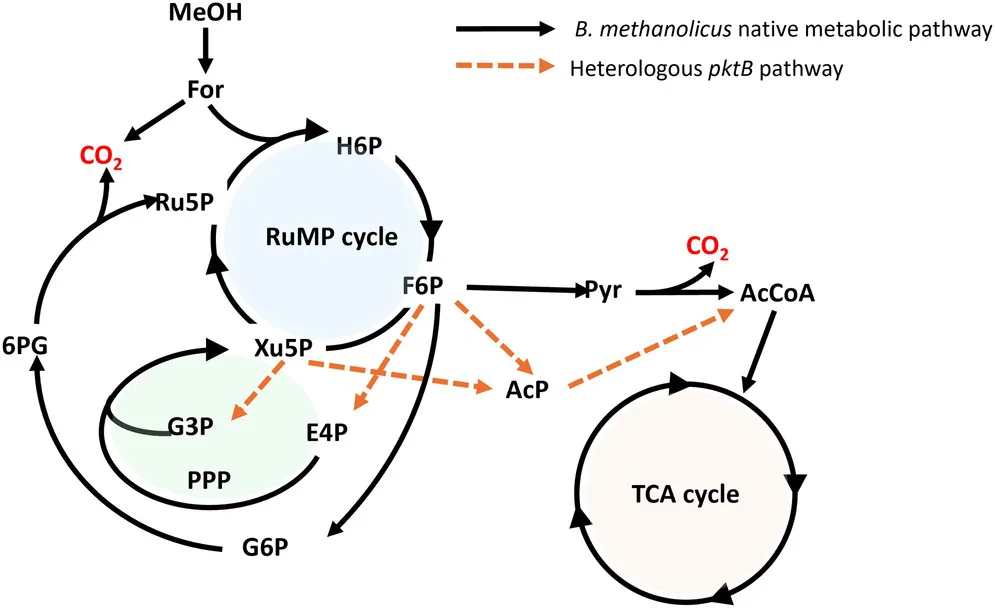
Central carbon metabolism of *Bacillus methanolicus* with heterologous phosphoketolase‐mediated cleavage reactions. Black arrows indicate native metabolic pathways, whereas orange dashed arrows indicate heterologous phosphoketolase‐mediated cleavage of fructose‐6‐phosphate to erythrose‐4‐phosphate and acetyl‐phosphate, and xylulose‐5‐phosphate to glyceraldehyde‐3‐phosphate and acetyl‐phosphate. Metabolite abbreviations: MeOH, methanol; For, formaldehyde; H6P, hexose‐6‐phosphate; F6P, fructose 6‐phosphate; Xu5P, xylulose 5‐phosphate; Ru5P, ribulose 5‐phosphate; Pyr, pyruvate; AcCoA, acetyl‐CoA; AcP, acetyl‐phosphate; G3P, glyceraldehyde 3‐phosphate; E4P, erythrose 4‐phosphate; G6P, glucose 6‐phosphate; 6PG, 6‐phosphogluconate. Pathway abbreviations: RuMP: Ribulose monophosphate; PPP: Pentose phosphate pathway; TCA: Tricarboxylic acid.

Methylotrophic conversion of single carbon (C1) feedstocks to fuels and industrial chemicals is an emerging component of the bioeconomy (Cotton et al. 2020; Sauvageau et al. 2024). During growth on methanol, a substantial fraction of assimilated carbon is ultimately released as biogenic CO_2_ through central metabolic reactions, including pyruvate decarboxylation to acetyl‐coA (AcCoA) and the oxidative branch of the RuMP cycle. This carbon loss limits biomass yields and remains a major challenge for industrial methylotrophic bioprocesses. High‐yield bioconversion technologies that minimize carbon loss present a path to overcome these hurdles and accelerate deployment of C1 microbial platforms. Pyruvate decarboxylation, a key branch point during substrate assimilation into central carbon metabolism, is a shared feature of nearly all conventional microbial platforms currently in use or under development (Bogorad et al. 2013), and reducing carbon loss at this metabolic junction is an established strategy to develop low oxidative, carbon‐efficient biocatalysts. Indeed, previous work has demonstrated that bypass of pyruvate decarboxylation can be achieved via utilization of a phosphoketolase (PKT)‐based carbon‐conserving pathway, enabling dramatic enhancement in biomass yield and carbon conversion efficiency across multiple microbial hosts (Henard et al. 2017; Henard et al. 2015; Yevenes and Frey 2008; Meile et al. 2001). PKT catalyses the cleavage of either fructose‐6‐phosphate (F6P) or xylulose‐5‐phosphate (X5P) into acetyl‐phosphate and either erythrose‐4‐phosphate (E4P) or glyceraldehyde‐3‐phosphate (G3P), respectively (Figure 1) (Henard et al. 2015). Acetyl‐phosphate (AcP) is converted to AcCoA either in a single step with phosphotransacetylase, or in two steps with acetate kinase followed by AcCoA synthetase. Because acetyl‐phosphate can subsequently be converted to AcCoA without pyruvate decarboxylation, this route provides a carbon‐conserving alternative for AcCoA biosynthesis. Meanwhile, the two side products E4P and G3P are recycled into the RuMP/pentose phosphate pathways. By bypassing pyruvate decarboxylation, incorporation of the PKT pathway has the potential to improve carbon conservation during methylotrophic growth.

The ribulose monophosphate (RuMP) cycle, a C1 assimilation pathway in many methylotrophic bacteria, has several intermediate molecules (including E4P and X5P) in common with the pentose phosphate pathway and PKT pathway (Orita et al. 2006). Furthermore, methylotrophic organisms that utilize the RuMP cycle often direct significant quantities of substrate carbon through the pathway, including the intermediates F6P and X5P (de la Torre et al. 2015; Akberdin et al. 2018; Delepine et al. 2020). Therefore, methylotrophic, RuMP‐utilizing organisms are well poised to reap the full benefits of PKT‐mediated carbon conversion. Indeed, we have previously shown that the methanotroph *Methylotuvimicrobium buryatense* 5GB1C exhibited substantial increases in methane conversion efficiencies when its native *pktB* gene was overexpressed (Henard et al. 2017).

*Bacillus methanolicus* is a thermophilic facultative RuMP methylotroph capable of utilizing methanol as a sole carbon and energy source (Heggeset et al. 2012). This organism is of particular interest for its ability to produce large quantities of glutamate, with titers reaching > 50 g L^−1^ under optimized conditions, and lysine (Schendel et al. 2000; Brautaset et al. 2007; Irla and Wendisch 2022; Schendel et al. 1990). Furthermore, *B. methanolicus* exhibits a rapid doubling time (1.4 h) under optimal conditions when growing on methanol, exceeding other native methylotrophs such as *Methylobacterium extorquens AM1* (3.6 h) and *Pichia pastoris* (4.6 h), as well as engineered *Escherichia coli* strains (4.3 h) (Yang et al. 2025; Reiter et al. 2024). While *B. methanolicus* does not contain a native *pkt* gene, it does encode the requisite genes for downstream conversion of AcP to AcCoA. *B. methanolicus* is therefore a suitable test chassis for demonstrating PKT‐mediated reductions in carbon loss. However, it was unclear whether previously observed phenotypes could be seen with the incorporation of PKT into a methylotrophic organism that does not harbour native PKT‐encoding genes.

Herein, we report PKT‐based engineering of *B. methanolicus* MGA3 to enhance biomass yield through incorporation and expression of the *pktB* gene from *M. buryatense* . We developed a suite of gene expression tools to first evaluate inducible and constitutive expression of PKT *in B. methanolicus*, then integrated *pktB* into the chromosome using homologous recombination. Resulting strains display significantly enhanced methanol‐to‐biomass yields, and mechanistic evaluation revealed that yield enhancements are concomitant with reduction in biogenic CO_2_ production. These data demonstrate the broad utility of PKT technology in C1‐utilizing microbes, establish a platform for basic science inquiry of carbon partitioning, and present a promising chassis for further applied industrial development.

## 2 Materials and Methods

### 2.1 Microbial Strains, Growth Media and Routine Culture Conditions

Unless otherwise described, *B. methanolicus* strains (Table S1) were routinely cultured at 42°C for all growth, fluorescence characterization, and biomass yield experiments presented in this study. *Bacillus methanolicus* was routinely cultured in MVcM media containing 235 mM K_2_HPO_4_, 108 mM NaH_2_PO_4_, 160 mM (NH_4_)_2_SO_4_, 10 mM MgSO_4_. 1 mL of vitamin metal stock solution (410 μM d‐biotin, 330 ΜM thiamine HCl, 270 μM riboflavin, 590 μM pyridoxine HCl, 460 μM pantothenate, 820 μM nicotinamide, 150 μM p‐aminobenzoic acid, 20 μM folic acid, 10 μM vitamin B12, 50 μM lipoic acid). 1 mL of trace metal stock (20 mM FeSO_4_, 160 μM CuCl_2_, 50 mM CaCl_2_, 170 μM CoCl_2_, 50 mM MnCl_2_, 1 mM ZnSO_4_, 200 μM Na_2_MoO_4_, and 500 μM H_3_BO_3_) was added after sterilization of 1 L basal medium in autoclave. Kanamycin was added to appropriate cultures at 50 μg mL^−1^. All media was pre‐warmed to 42°C before inoculation. Cultures were routinely activated from the −80°C freezer via inoculation with permanent stocks and cultured in baffled shake flask in MVcM amended with 0.25 g L^−1^ yeast extract (MVcMY). Methanol was amended at 200 mM immediately prior to inoculation. Stellar cells (Takara Bio USA Inc), an *Escherichia coli* HST08 strain, were grown in lysogeny broth (LB) amended with 50 μg mL^−1^ kanamycin at 37°C for plasmid construction.

### 2.2 Molecular Cloning and Plasmid Construction

All plasmid construction and molecular cloning were performed in *E. coli* Stellar cells (Takara Bio USA Inc). Plasmids were assembled using In‐Fusion Snap Assembly Master Mix (Takara Bio). DNA fragments were synthesized by Twist Bioscience (San Francisco, CA) with appropriate homology regions for In‐Fusion assembly. All plasmid sequencing was performed by Plasmidsaurus (Louisville, KY) using Oxford Nanopore Technology with custom analysis and annotation. Plasmids and primers used in this study are listed in Tables S2 and S3.

Construction of the anhydrous tetracycline (aTc) inducible plasmid was based on a previous report (Kamionka et al. 2005). The *Bacillus/E. coli* shuttle vector pUB110S (Irla et al. 2016) was slightly modified by replacing the neomycin resistance gene with the *ahp* Kan^R^ gene to create the pYT1 backbone used throughout this study (Kamionka et al. 2005). A previously described synthetic hybrid promoter consisting of the *B. subtilis xylA* promoter fused to the *Tn10 tet* operator (*p*_*xyl/tet*_) was selected as a candidate inducible expression system for *B. methanolicus* and assembled into pYT1. The *tetR* gene from bacterial transposon Tn*10* was subsequently added downstream of *p*_*xyl/tet*_ under control of the constitutive *B. methanolicus* methanol dehydrogenase promoter *p*_*mdh*_ to generate the aTc‐inducible expression vector termed pLM (Tables S2 and S3).

To characterize inducible expression in *B. methanolicus* , *sfGFP* was initially cloned downstream of p_*xyl/tet*_ to generate pLM‐*sfGFP*. Following characterization of the inducible system, the *Methylotuvimicrobium buryatense pktB* gene (EQU24_07895), codon optimized for *B. methanolicus* , was substituted in place of *sfGFP* to generate pLM‐*pktB*.

To evaluate constitutive expression, several native *B. methanolicus* promoter regions were selected for evaluation including dipeptide‐binding protein (*dppE*; BMMGA3_08820), pyridoxine kinase (*pdxK*; BMMGA3_16310, Irla et al. 2021), isocitrate dehydrogenase (*icd*; BMMGA3_13055), and elongation factor tu (*tuf*; BMMGA3_00685). The 200‐bp region immediately upstream of each selected gene was synthesized by Twist Bioscience, with *sfGFP* immediately downstream of a ribosome binding site (AGGAGG, Irla et al. 2015) to generate the constitutive expression vectors pYT‐*dppE‐sfGFP*, pYT‐*icd‐sfGFP*, pYT‐*pdxK‐sfGFP*, and pYT‐*tuf‐sfGFP*. Following promoter characterization, the corresponding *sfGFP* coding sequence was replaced with codon‐optimized *pktB* to generate the constitutive expression vectors pYT‐*dppE‐pktB*, pYT‐*pdxK‐pktB*, pYT‐*icd‐pktB*, and pYT‐*tuf‐pktB*.

Finally, a chromosomal integration vector was constructed following the temperature‐sensitive integration strategy recently described for *B. methanolicus* (Li et al. 2025). Briefly, pYT1 was modified by inserting *sfGFP* downstream of *p*_*mdh*_ between *repB* and the temperature‐sensitive origin of replication to generate pYT21. Homology arms targeting the genomic region immediately upstream of the purine biosynthesis gene cluster (beginning at BMMGA3_RS01640) were amplified from *B. methanolicus* genomic DNA and assembled with the *pdxK‐pktB* expression cassette to generate the integration vector pYT31.

### 2.3 *Bacillus methanolicus* Electroporation

Plasmids were electroporated into *B. methanolicus* following a previously described protocol (Jakobsen et al. 2006). First, electrocompetent cells were prepared by inoculating 5 mL of SOB supplemented with 0.25 M sucrose (SOB_suc_) with a freezer stock of wild type *B. methanolicus* MGA3 and grown overnight at 50°C. A 5 mL aliquot of this overnight culture was used to inoculate 100 mL of pre‐warmed SOB_suc_ and grown at 50°C until OD_600_ ~ 0.25. Cells were harvested immediately by centrifugation at 4000 x *g* for 5 min at 4°C and washed twice in 10 mL EP buffer containing 1 mM HEPES buffer at pH 7.0 and 25% v/v polyethylene glycol 8000. Washed cells were resuspended in 0.5 mL of cold EP buffer, partitioned into 100 μL aliquots and frozen at −80°C until use. To begin electroporation, cells were thawed on ice, and 0.5–1 μg of plasmid DNA was added to each tube. Cells were incubated on ice for 30 min with DNA before being pulsed at 2.5 kV; 200 Ohms; 25 μF with a Bio‐Rad Gene Pulser in a pre‐chilled 2 mm gap cuvette. The cells were immediately transferred to 5 mL of prewarmed SOB_suc_ and incubated overnight at 50°C. The whole culture volume was then inoculated into 50 mL of pre‐warmed SOB_suc_ containing kanamycin and grown at 50°C for 6–8 h. Cells were then pelleted and spread onto prewarmed SOB_suc_‐kanamycin agar and incubated at 50°C overnight. Finally, single colonies were inoculated into prewarmed selective MvCMY medium amended with 200 mM methanol and grown overnight at 42°C with 250 rpm shaking. When the OD reached ~2.0–2.5 the next morning, cultures were immediately transferred for long‐term storage in a −80°C freezer in 10% v/v DMSO.

### 2.4 Characterization of the aTc‐Inducible Cassette pLM in *B. methanolicus*

Due to the potential toxicity of aTc, dose‐dependent expression of *sfGFP* was assessed to determine the optimal aTc concentration for future *pktB* induction. Following inoculation at OD_600_ ~ 0.1, 0–1.2 μM aTc was added to *B. methanolicus* pLM‐*sfGFP* cultures in triplicate once they doubled to OD_600_ ~ 0.2, and cell growth and fluorescence were monitored by measuring cell density at 600 nm and sfGFP fluorescence at 485 nm excitation/535 nm emission using a Tecan fluorometer. 0.8 μM aTc was selected as the optimal working concentration for pLM induction by comparing maximal relative fluorescent unit (RFU) values (normalized to cell density) and growth rates. A similar experiment was conducted with both the empty pLM control and pLM‐*sfGFP* strains, amended both with and without 0.8 μM aTc, and cell growth and fluorescence were monitored for 24 h to further characterize the *sfGFP* expression profile (Figure S1b). A final test was conducted to determine how aTc depletion in culture media would affect fluorescence: following inoculation at OD_600_ ~ 0.1, 0.4 μM of aTc was delivered to triplicate *B. methanolicus* pLM‐*sfGFP* cultures in three doses at OD_600_ 0.2, 0.4, and 0.8, and cell growth and fluorescence signals were monitored.

### 2.5 Evaluation of aTc‐Induced pktB Expression and Biomass Enhancement in *B. methanolicus*

Following validation of the pLM cassette in *B. methanolicus* , the pLM‐*pktB* strain was deployed to determine whether *M. buryatense* *pktB* confers a biomass yield enhancement to *B. methanolicus* when cultured with methanol. The pLM and pLM‐*pktB* strains (Table S1) were activated and cultured as previously described in 2.1 at a 50 mL culture volume in triplicate 125 mL baffled flasks. 0.8 μM aTc was amended to each flask following cell density doubling to OD_600_ ~ 0.2. At regular intervals, 1 mL culture was removed for cell density determination and then filtered using a 0.22 ΜM filter to later measure methanol consumption via HPLC. Once cultures reached OD_600_ ~ 2.0, cells were harvested by centrifugation and stored at −80°C prior to lyophilization. Cell pellets were first lyophilized in a FreeZone 2.5 L freeze dryer (Labconco, Missouri, USA) and weighed to obtain dry cell weight. Biomass enhancements were calculated by comparing the grams of dry cell weight per gram of methanol consumed and normalized to the empty vector control strain pLM.

### 2.6 Evaluation of Several Constitutive Native *B. methanolicus* Promoters

Each constitutive *sfGFP*‐expressing strain (Table S1) was activated and precultured as described in 2.1 and inoculated between OD_600_ 0.1–0.2 into triplicate 125 mL baffled flasks containing MvCM media amended with 200 mM methanol. Cell growth and fluorescence were monitored by measuring cell density at 600 nm and sfGFP fluorescence at 485 nm excitation/535 nm emission using a Tecan fluorometer (Irla et al. 2015, 2021).

### 2.7 Evaluation of pktB‐Mediated Biomass Enhancements in *B. methanolicus*

Following standard activation and preculture, constitutive *pktB*‐expressing strains (Table S1) were inoculated in standard conditions between OD_600_ 0.1–0.2 into triplicate 125 mL baffled flasks containing MvCM media amended with 200 mM methanol. At regular intervals, 1 mL of culture was removed for cell density determination and then filtered using a 0.22 μM filter to later measure methanol consumption via HPLC. Once cultures reached OD_600_ ~ 2.0, cells were harvested by centrifugation and stored at −80°C until lyophilization. Cell pellets were first lyophilized in a FreeZone 2.5 L freeze dryer (Labconco, Missouri, USA), then biomass enhancements were calculated by comparing the grams of dry cell weight per gram of methanol consumed and normalized to the empty vector control strain YT1 or wild type strain. The strain that displayed the highest biomass enhancement without a growth defect, *pdxK‐pktB*, was down‐selected for further analysis.

### 2.8 Phosphoketolase Enzyme Assay

The pYT1 empty vector and *pdxK‐pktB* strains were grown under standard conditions with methanol to further confirm *pktB* activity in *B. methanolicus* from whole‐cell lysates. 10^9^ cells were harvested in triplicate from cultures in mid‐log phase (OD_600_ ~ 2.0) by centrifugation at 4°C and 4000 *g* for 10 m. Cell pellets were resuspended in 100 μL 50 mM potassium phosphate buffer (pH 6.5) and disrupted by sonication on ice at an amplitude of 55% and a duty cycle of 0.5 for 9 min with a 30 s pause between each cycle (López et al. 2019). Whole‐cell lysates were centrifuged for 1 h at 4°C and 12,100 × *g*. The enzymatic assay was performed following previous reports (Henard et al. 2017; Glenn and Smith 2015). Reaction mixtures (100 ΜL total volume) comprised of 30 mM potassium phosphate (pH 6.5), L‐cysteine hydrochloride (2 mM), sodium fluoride (20 mM), sodium iodoacetate (10 mM), thiamine pyrophosphate (1 mM), and fructose‐6‐phosphate (30 mM) were initiated by the addition of 25 μL (10 μg total protein) of whole‐cell extracts. Reactions were incubated at 42°C for 1 h before 50 μL of 2 M hydroxylamine hydrochloride (pH 7.0) was added to the reaction and incubated at room temperature for 10 min. Reactions were then terminated by adding 300 μL of a 1:1 mixture of 2.5% ferric chloride in 2 M hydrochloric acid and 10% trichloroacetic acid. A colour change due to ferric‐hydroxamate formation was measured at 505 nm using a Tecan fluorometer, and the concentration of AcP formed during the initial reaction was calculated by regression analysis compared to known standards.

### 2.9 Carbon Dioxide Production by pktB‐Expressing Strains

To determine whether CO_2_ production was decreased in *pktB*‐expressing strains, cultures were grown in stoppered 500 mL Duran Pressure Plus bottles (DWK Life Sciences, USA) filled with 10 PSI of compressed air. Each strain was inoculated at OD ~0.2 into 50 mL of MvCM amended with 200 mM methanol. Bottles were incubated with shaking at 250 rpm until they reached OD ~2.0. At this point, 1 mL of 2 M sulfuric acid was added in excess to each bottle to end the experiment and liberate all dissolved CO_2_. 50 μL of headspace gas was measured by gas chromatography and compared to an external calibration curve. Headspace gas was injected into an Agilent 7890A gas chromatograph using a thermal conductivity detector and a Porapak N column.

### 2.10 Integration of *pktB* Into the *B. methanolicus* Chromosome and Evaluation of Biomass Enhancement in Resultant Integrated Strain

The recent development of a chromosomal integration method for *B. methanolicus* enabled us to determine whether the biomass yield enhancement conferred by plasmid‐based *pktB* expression could be maintained following genomic integration. The recently reported integration method utilizes a temperature‐sensitive replication origin to force chromosomal integration via homologous recombination (Li et al. 2025). Following electroporation with pYT31 (Section 2.2), bright green single colonies were streaked onto selective agar plates and incubated at 60°C. After 2–3 days, some of the colonies exhibited weaker fluorescent signals under a blue light, indicating a single‐crossover event. Single crossover colonies were inoculated into SOB medium (nonselective), cultured overnight at 50°C, then passaged 1–2 additional times at 60°C. The whole culture volumes were centrifuged, plated on SOB medium, then incubated at 50°C. Colonies were subsequently patched dually onto both SOB medium and selective SOB medium, and colonies that did not grow on selective medium (which also did not display any fluorescent signal) were subsequently down‐selected for PCR verification of integration (Table S3), and the resulting strain was termed BMGA3::*pktB* (Table S1).

The BMGA3::*pktB* strain was characterized in a similar manner to the plasmid‐based strains using the wild type as a control. Methanol utilization, cell growth, and biomass enhancement from methanol were determined in a manner identical to the method described in 2.7. For later RNA isolation and reverse‐transcriptase PCR (RT‐PCR), 10^9^ cells from each flask were resuspended in the appropriate volume of DNA/RNA shield (Zymo Research) during mid‐log phase growth, centrifuged at 4°C and decanted, immediately flash‐frozen in liquid nitrogen and stored at −80°C. Biogenic CO_2_ production was determined following the method previously described in Section 2.9.

### 2.11 Metabolite Quantitation

High performance liquid chromatography (HPLC) was used to determine methanol and acetate concentrations. Samples were loaded onto an Agilent 1260 Infinity II system equipped with the Agilent 1260 Infinity II Series Refractive Index Detector. LC separation was performed using an Aminex HPX‐87H chromatographic column with ionic form H+/CO3‐deashing guard column at 55°C. 4 mM sulfuric acid aqueous solution was used as the mobile phase. The flow rate was 0.6 mL min^−1^ and the injection volume was 50 μL per sample. Metabolite concentrations were measured by comparing samples to known standards.

### 2.12 RNA Isolation and Reverse Transcription‐PCR

RNA isolation was performed according to the manufacturer instructions with the RNeasy Mini Kit (QIAGEN, Hilden, Germany). The Quick‐Start Protocols were followed, RNAprotect Bacteria Reagent, RNeasyProtect Bacteria Kits and RNeasyMini Kit, Part 1 and Part 2. For the on‐column DNase digestion, RNase‐Free DNase Set (QIAGEN, Hilden, Germany) was used according to the manufacturer instructions. RNA quality and quantity were determined utilizing a Qubit 4 Fluorometer (Thermo Fisher Scientific, Massachusetts, USA). RT‐PCR was executed on RNA using iTaq Universal SYBRGreen One‐Step Kit (Bio‐Rad Laboratories, California, USA) according to the manufacturer instructions. 100 ng of RNA was used per reaction and primers were used at a concentration of 200 nM. Primer pairs for the *rpoB* housekeeping gene and *pktB* gene are listed in Table S3. The comparative Ct (ΔΔCt) experiment type was used. Cycling conditions were run at 50°C for 10 min, 95°C for 1 min, 95°C for 15 s, 60°C for 1 min for 40 cycles. Carboxyrhodamine was used as the passive reference dye. RT‐PCR was performed on QuantStudio 3 System (Applied Biosystems, Massachusetts, USA). Relative quantification was calculated using the Pfaffl method.

## 3 Results

### 3.1 Optimization of Inducible and Constitutive Expression Systems in *B. methanolicus*

**FIGURE 2:**
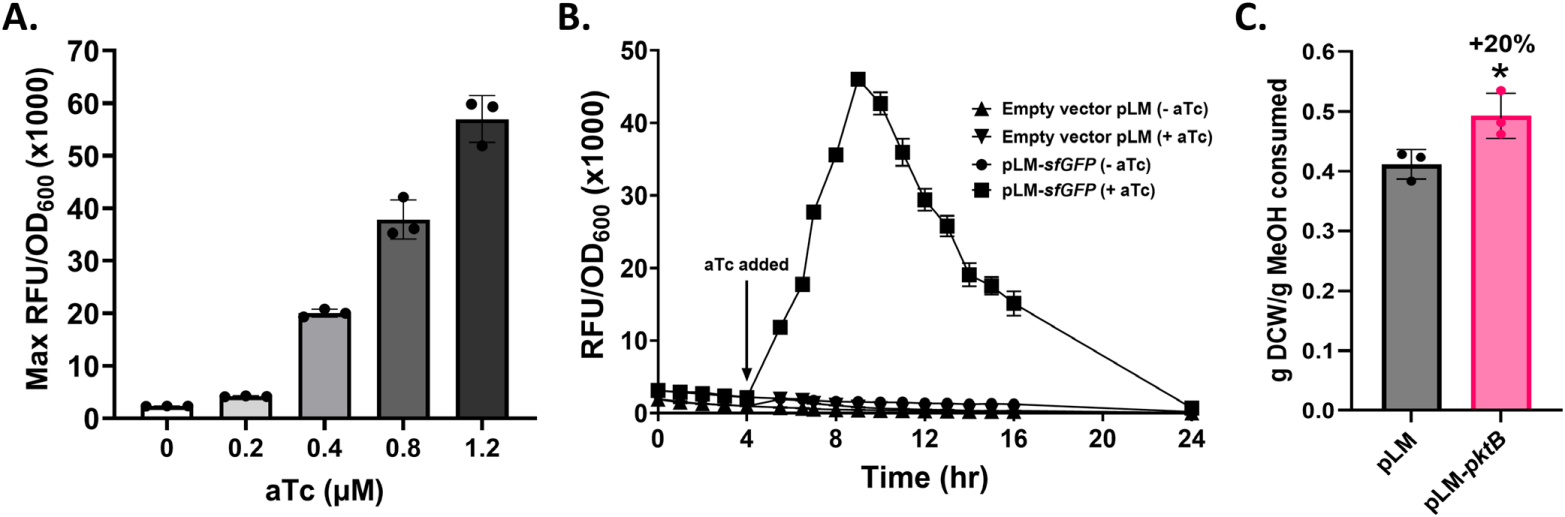
aTc inducible expression cassette is tunable and effectively expresses *pktB*. (A) Relative expression levels of sfGFP as the aTc concentration is increased. (B) Relative expression levels of sfGFP over time after aTc addition. Error bars show (SEM) for 3 replicates. (C) Grams of dry cell weight per gram methanol consumed for the pLM control and pLM‐*pktB* strains. Error bars show standard error of the mean (SEM) for 3 replicates. *p < 0.05.

A previously modified *B. subtilis* *xylA* promoter that contained the Tn*10 tet* operator site, named *p*_*xyl/tet*_, was selected as a candidate for inducible gene expression in *B. methanolicus* (Kamionka et al. 2005) (Jakobsen et al. 2006; Brautaset et al. 2004). The *p*_*xyl/tet*_ promoter was initially fused with *sfGFP* as a reporter gene to characterize *p*_*xyl‐tet*_ in *B. methanolicus*, and induced with 0–1.2 μM anhydrous tetracycline (aTc). Maximum RFU values (normalized to cell density) and growth rates were compared between concentrations (Figure 2a, Figure S1a). *sfGFP* expression was induced by all tested concentrations of aTc; however, a growth defect was displayed in the cultures amended with 1.2 μM aTc (Figure S1a), so 0.8 μM aTc was selected for optimal induction for subsequent experiments.

The temporal *sfGFP* expression profile following induction with 0.8 μM aTc was then evaluated (Figure 2b). Over the first 5 h following aTc addition (corresponding to approximately 9 h on the experimental time course), sfGFP fluorescence increased approximately 19‐fold, from ~2450 RFU OD_600_^−1^ to ~46,000 RFU OD_600_^−1^. Thereafter, fluorescence normalized to cell density gradually declined, likely due to depletion of free aTc, which binds TetR with high affinity (Aleksandrov et al. 2009). Consistent with this interpretation, repeated addition of aTc resulted in continued increases in maximum sfGFP fluorescence over time (Figure S1c), whereas a single aTc dose produced a maximum fluorescence of approximately 46,000 RFU OD_600_^−1^ (Figure 2b). In contrast, both the empty vector control (pLM) and the uninduced pLM‐*sfGFP* strain maintained low fluorescence throughout growth, demonstrating minimal background *sfGFP* expression (Figure 2b).

**FIGURE 3:**
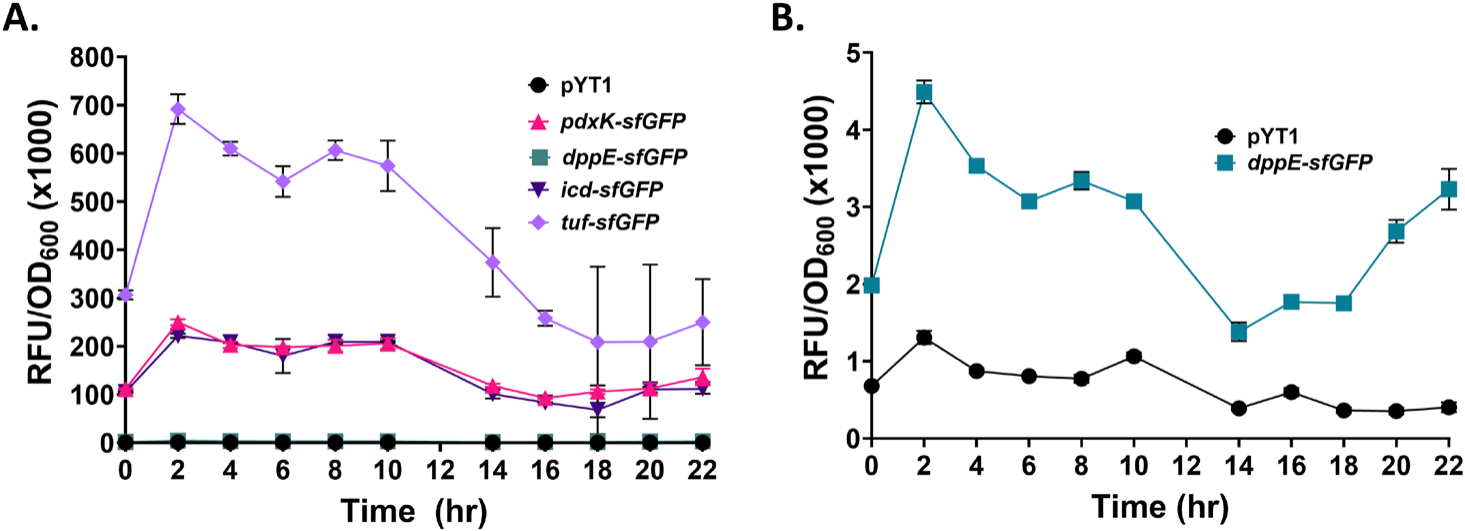
sfGFP expression over time using native constitutive promoters. (A) Relative expression of sfGFP over time for *dppE*, *pdxK*, *icd*, and *tuf* sfGFP expression vectors. (B) Relative sfGFP expression over time for the dppE expression vector. Panel B presents the same dataset shown in panel A using a different y‐axis scale to better visualize the relatively low expression driven by the dppE promoter. Error bars represent the SEM from three biological replicates.

The temporal sfGFP expression profile indicated that a single dose of aTc sustained gene induction for approximately 5 h before inducer depletion reduced expression (Figure 2b). Thus, constitutive expression was evaluated next, and several putative constitutive promoters of varying strengths were identified. Selected promoters included *dppE*, *pdxK*, *icd*, and *tuf*. The promoters were initially used to drive expression of *sfGFP* to determine their relative strengths and confirm a constitutive expression profile (Figure 3a,b). The *tuf* promoter displayed the highest relative *sfGFP* expression levels (691,000 RFU OD_600_^−1^), followed by *pdxK* (250,000 RFU OD_600_^−1^) and *icd* (222,000 RFU OD_600_^−1^), and finally *dppE* (4500 RFU OD_600_^−1^). In all strains, *sfGFP* fluorescence reached its maximum during early log phase growth, then slowly decreased to a stable level of expression into late log and stationary phase (Figure 3a,b). Compared with the empty vector strain, all constitutive *sfGFP* strains displayed a moderate to severe growth defect (Figure S3a).

### 3.2 Induced Expression of pktB Enhances Methanol to Biomass Yield by *B. methanolicus*

Because phosphoketolase expression had not previously been evaluated in *B. methanolicus* , we first examined an aTc‐inducible expression system to provide a controllable means of assessing *pktB* expression before constructing constitutive *pktB* expression strains. The pLM‐*pktB* strain was compared to the empty vector control pLM using the previously‐established induction scheme (see Section 2.5). After aTc induction, cultures were subsequently left to grow to OD_600_ ~ 2.0 (Figure S2), at which point biomass was harvested and methanol consumption determined. The pLM‐*pktB* strain displayed a 20% increase in dry cell weight biomass per gram of methanol consumed relative to the empty pLM control (0.41 ± 0.02 versus 0.49 ± 0.04 g DCW g^−1^ MeOH^−1^ consumed, respectively) (Figure 2c).

### 3.3 Constitutive Expression of *pktB* Further Enhances Methanol‐To‐Biomass Yield in *B. methanolicus*

**FIGURE 4:**
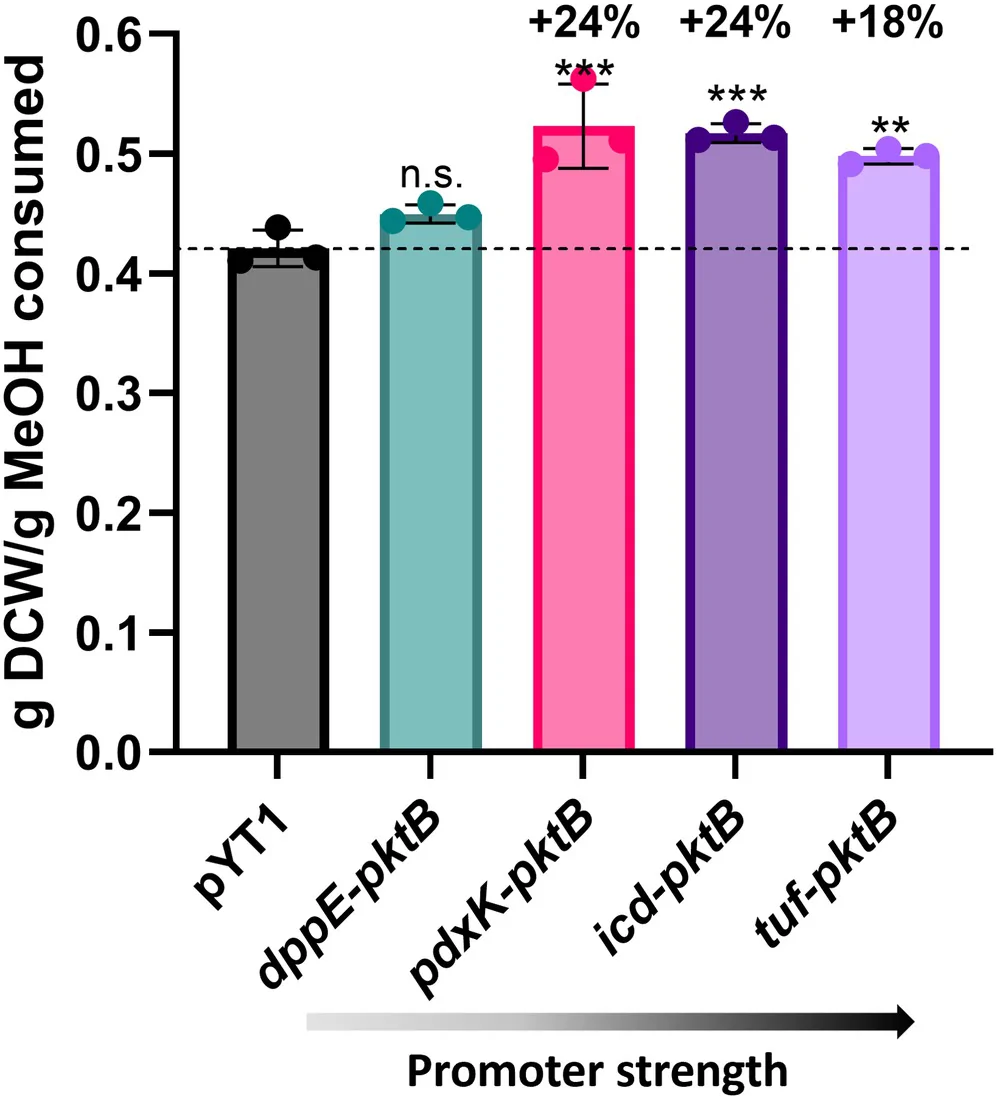
Methanol‐to‐biomass yield enhancements by constitutive *pktB*‐expressing strains. Grams of dry cell weight yielded per gram methanol consumed for each strain tested. Error bars show SEM for 3 replicates. ** *p* < 0.01, *** *p* < 0.001. ‘n.s.’ was no significance.

When *pktB* was expressed under constitutive promoters, all strains demonstrated either similar or a growth defect when compared to the empty strain (Figure S3b). However, expression of *pktB* led to a biomass yield enhancement in the *pdxK‐pktB* (0.52 ± 0.04 g DCW g^−1^ MeOH^−1^ consumed), *icd‐pktB* (0.52 ± 0.01 g DCW g^−1^ MeOH^−1^ consumed), and *tuf‐pktB* (0.50 ± 0.01 g DCW g^−1^ MeOH^−1^ consumed) strains when compared to the empty vector strain baseline (0.42 ± 0.02 g DCW g^−1^ MeOH^−1^ consumed) (Figure 4). In contrast, the results of the *dppE‐pktB* strain (0.45 ± 0.08 g DCW g^−1^ MeOH^−1^ consumed) were not significantly different from the control. The *pdxK‐pktB* strain, which displayed the greatest biomass yield enhancement, was subsequently down‐selected for further characterization.

### 3.4 PktB Is Functionally Active in *B. methanolicus* and Reduces Biogenic CO_2_ Production

**FIGURE 5:**
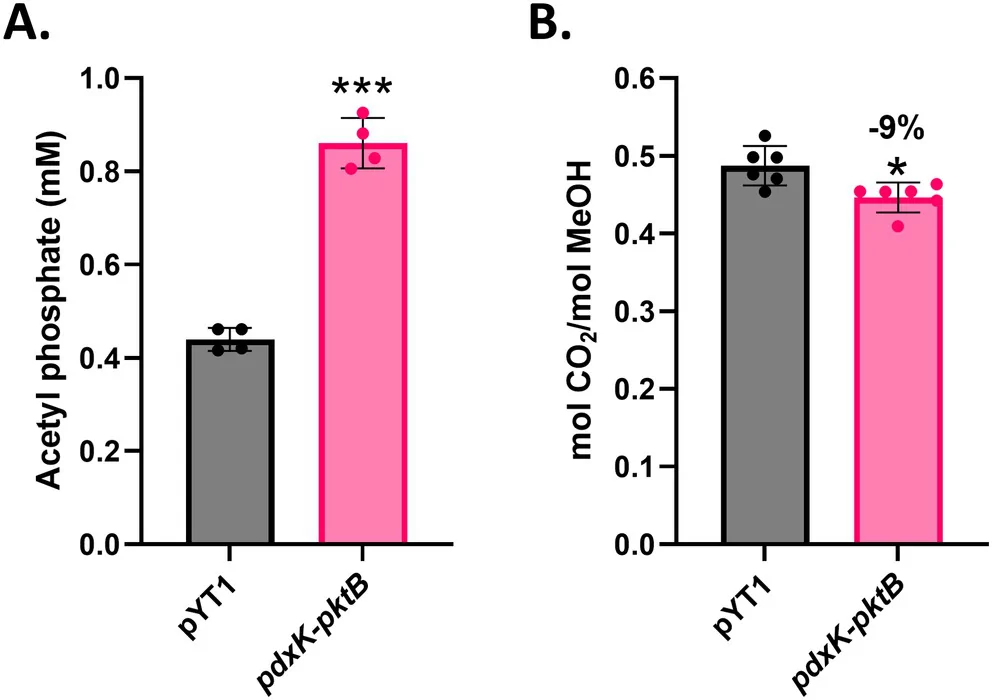
Methanol‐to‐biomass yield enhancements by the constitutive *pdxK‐pktB* strain. (A) Hydroxymate phosphoketolase activity assay indicating AcP concentrations normalized to cell density. (B) relative mol of CO_2_ produced per mol of methanol consumed. Error bars show SEM for 4 replicates for the phosphoketolase activity assay and 6 replicates for CO_2_ production. * *p* < 0.05. *** *p* < 0.001.

To determine whether the PktB enzyme was indeed active in *B. methanolicus* , whole‐cell lysates from the *pdxK‐pktB* strain were analysed via a hydroxymate assay using the concentration of AcP, a product of PktB catalysis (Figure 1), as a readout for PKT activity. Normalized to cell density, the empty control strain YT1 produced 0.44 ± 0.02 mM AcP (Figure 5a) while the *pdxK‐pktB* strain produced 0.86 ± 0.05 mM AcP, an almost 2‐fold increase, indicating PktB was functionally active in *B. methanolicus* . Next, RNA was analysed via RT‐PCR to quantify *pktB* expression in the *pdxK‐pktB* strain. During mid‐log growth, *pktB* was detected at 13.13 ± 0.74 times the control housekeeping gene *rpoB*, indicating high relative expression (Figure S4).

Since PKT catalysis bypasses pyruvate decarboxylation directly, and other CO_2_ production nodes indirectly, we hypothesized that *pktB* activity in *B. methanolicus* should lead to decreased CO_2_ production during growth on methanol. To test this hypothesis, the empty vector control and the *pdxK‐pktB* strains were grown inside sealed pressure bottles, and the amount of CO_2_ produced was determined via GC analysis once cultures reached an OD_600_ ~ 2.0. The empty vector control strain YT1 produced 0.49 ± 0.02 mol CO_2_ mol^−1^ MeOH^−1^ consumed, while the *pdxK‐pktB* strain produced 0.44 ± 0.02 mol CO_2_ mol^−1^ MeOH^−1^ consumed, representing a 9% reduction in total CO_2_ production from the *pktB*‐expressing strain (Figure 5b).

### 3.5 Genomic Integration of pktB Retains Methanol to Biomass Yield Enhancements and Reduces Biogenic CO_2_ Production

**FIGURE 6:**
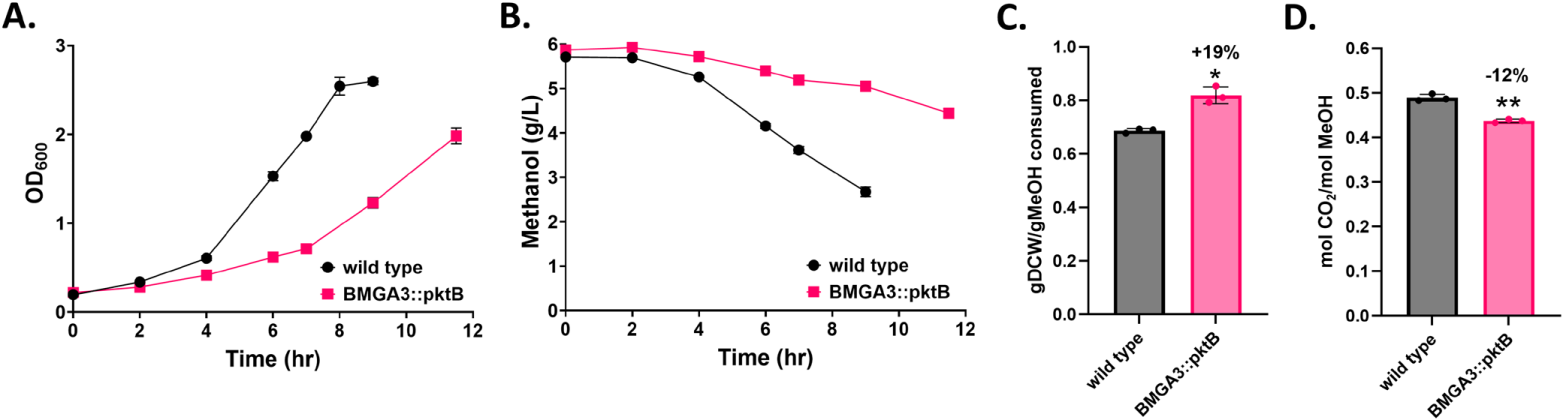
Characterization of growth, methanol consumption, biomass yield, and CO_2_ production of the wild type vs BMGA3::pktB strains on methanol. (A) Optical density over time. (B) Methanol consumption by each strain over time. (C) Grams of dry cell weight yielded per gram methanol consumed for each strain. (D) Relative mol of CO_2_ produced per mol of methanol consumed. Error bars show SEM for 3 replicates in all experiments. * *p* < 0.05, ** *p* < 0.01.

Following confirmation of the integrated *pktB* strain BMGA3::*pktB*, RNA was analysed via RT‐PCR to confirm and quantify *pktB* expression in the BMGA3::*pktB* strain. During mid‐log growth, *pktB* was detected at 0.16 ± 0.03 times the control housekeeping gene *rpoB*, indicating expression (Figure S4). Next, cell growth and biomass yield from methanol was tested following the same methods described previously. A growth defect was observed in the BMGA3::*pktB* strain compared with the wild type (Figure 6a). Consistent with this phenotype, the integrated strain exhibited a slower methanol assimilation rate than the wild type (Figure 6b). Despite this reduction in substrate consumption rate, expression of *pktB* in the integrated BMGA3::*pktB* strain led to a biomass yield enhancement of 19% (0.82 ± 0.03 g DCW g^−1^ MeOH^−1^ consumed) when compared to the wild type strain (0.69 ± 0.01 g DCW g^−1^ MeOH^−1^ consumed) (Figure 6c).

The same CO_2_ quantification experiment described in Section 2.9 was repeated for the wild type and integrated BMGA3::*pktB* strains. The BMGA3::*pktB* strain produced 0.44 ± 0.01 mol CO_2_ mol^−1^ MeOH^−1^ consumed, while the wild type strain produced 0.49 ± 0.004 mol CO_2_ mol^−1^ MeOH^−1^ consumed, representing a 12% reduction in total biogenic CO_2_ production displayed by the BMGA3::*pktB* strain (Figure 6d).

## 4 Discussion

Methylotrophic organisms have potential to be key contributors in the emerging bioeconomy by utilizing C1 compounds like methanol, methane, or methylamine as sole sources of carbon and energy to produce relevant platform chemicals for biomanufacturing. However, much like conventional heterotrophic biocatalysts, methylotrophs suffer from limited carbon conversion efficiency as significant carbon is lost through metabolic CO_2_ production, hindering their implementation in the bioeconomy. The results described in the present study highlight a strategy in which biocatalysts can be engineered to reduce carbon loss during growth by the addition of PKT, an enzyme capable of directly bypassing typical pyruvate decarboxylation and improving substrate‐to‐biomass conversion yield. Our chosen PKT was *pktB* from native *Methylotuvimicrobium buryatense* 5GB1C due to its ability to improve biomass yield from methane in previous studies (Henard et al. 2017). When this *pkt* was overexpressed in *M. buryatense* , a > 2‐fold biomass enhancement was observed during growth. To investigate whether this same enzyme would be functional and confer biomass enhancements in *B. methanolicus*, we first utilized an inducible expression cassette based on the tetracycline cassette from the *E. coli* Tn10 (Chalmers et al. 2000) to drive expression of *pktB*. We initially chose to inducibly express *pktB* as it was unknown whether *pktB* expression in *B. methanolicus* would be burdensome. This aTc cassette was previously reported for use in *B. subtilis* , which we optimized for *B. methanolicus* by adding a strong native promoter (*mdh*) to control *tetR* expression. In *B. methanolicus* , this cassette not only boasted high expression following induction and little background expression but was also tunable by varying inducer concentrations (Figure 2a). There are a limited number of inducible systems presently described for *B. methanolicus* , including a mannitol‐inducible system and a 2‐aminopurine riboswitch (Irla et al. 2016; Irla et al. 2021; Brito et al. 2025). However, each system has drawbacks: 2‐aminopurine can be costly compared to aTc, and mannitol is a native source of carbon and energy for *B. methanolicus* . The aTc‐inducible cassette presently described offers a cost‐effective and tunable alternative for gene expression. This system was deployed for both tunable *sfGFP* and *pktB* expression, improving biomass yields by 20% compared to the control strain (Figure 2). aTc induction is therefore a serviceable tool for inducible heterologous gene expression in *B. methanolicus* and expands the repertoire of available tools for gene induction in this organism; however, this strategy is limited by aTc lifetime.

Given the transient nature of aTc, we assessed the use of constitutive promoters to drive *pktB* expression. Several promoters were screened that we expected to have high, medium, and low expression levels based on prior transcriptomic analysis (Irla et al. 2015). All *sfGFP*‐expressing strains displayed fluorescent signals throughout growth (Figure 3a,b), confirming functional expression. Furthermore, all strains harbouring constitutive expression of *pktB* showed biomass yield enhancements from methanol. Notably, however, the medium‐to‐high expression strains (*pdxk‐pktB*, *icd‐pktB*, and *tuf‐pktB*) showed little variation in the degree of biomass enhancement (Figure 4), with values between 18% and 24% greater biomass yield compared with the empty vector strain. Similarly, the biomass yield enhancement of the pLM‐*pktB* strain under aTc induction was 20% greater than its empty vector strain (Figure 2c).

Maintaining the pLM and pYT plasmids led to growth defects in the empty vector strains (compared with the wild type) and strains expressing non‐catalytic proteins such as sfGFP (Figure 6, Figure S2). Until recently, no published methods for chromosomal integration in *B. methanolicus* were available, leaving plasmid‐encoded expression as the only way to introduce new genes. Herein, we deployed the methodology described by Li et al. to enable integration of *pktB* into the chromosome (Li et al. 2025). Encouragingly, the integrated strain still displayed biomass yield enhancements from methanol, albeit slightly less than the plasmid‐based strains. These results indicate that a single chromosomal integration of *pktB* confers sufficient activity to produce yield enhancements. In addition, the relative expression of *pktB* as quantified by RT‐PCR of the plasmid‐driven *pdxK‐pktB* strain vs. the integrated BMGA3::*pktB* strain (13.13 times the housekeeping gene vs. 0.16 times the housekeeping gene, Figure S4) indicate that *pktB* expression is not the limiting factor for biomass yield enhancement. These results suggest that once *pktB* expression exceeds a certain threshold (i.e., higher than the *dppE* promoter), there may be other post‐transcriptional regulatory elements which may cap the biomass yield enhancement at around 24%. Such elements could include PktB thermolability, lack of native protein chaperones, misfolding, or more likely, PKT substrate limitation (Figure 1).

We anticipated that the biomass enhancement in all *pktB*‐expressing strains was associated with a reduction in CO_2_ production. As mentioned, PktB cleaves F6P to E4P and AcP, while X5P is cleaved to G3P and AcP, which can then be converted to AcCoA without the need for pyruvate decarboxylation and thereby avoiding CO_2_ generation at the pyruvate decarboxylation step. (Figure 1). Our results show that both plasmid‐based and integrated *pktB* expression did indeed reduce CO_2_ production, as we observed a 9% and 12% reduction in CO_2_ output, respectively, compared to control strains. Although the reductions in CO_2_ production were directionally consistent with increased biomass yield, the magnitudes of these changes were not strictly proportional, suggesting that additional changes in carbon partitioning, maintenance burden, or central metabolic flux may also contribute to the observed phenotype. In addition to directly bypassing pyruvate decarboxylation, the reduction in CO_2_ production may also reflect altered flux through the dissimilatory and assimilatory branches of the RuMP cycle. Because RuMP cycle intermediates interface extensively with the pentose phosphate pathway, PKT activity has the potential to recycle carbon intermediates back into central metabolism while reducing carbon loss associated with AcCoA formation (Figure 1). For example, carbon entering the PKT pathway from F6P may undergo multiple rounds of carbon rearrangement and cleavage, increasing the theoretical yield of AcCoA while minimizing CO_2_ release relative to the native pathway. However, in wild‐type *B. methanolicus* , F6P regenerates ribulose‐5‐phosphate (Ru5P) via the cyclic dissimilatory branch of the RuMP cycle, first passing through 6‐phosphogluconate before liberating one CO_2_. Removing some flux of F6P through this pathway (via diversion through *pktB*) could account for some of the reduction in CO_2_ production that was observed in the integrated *pktB* strain. However, although promiscuous F6P activity has been observed, PKTs found in Type I methanotrophs (e.g., *pktB*) preferentially utilize X5P as a substrate (Henard et al. 2015; Yevenes and Frey 2008; Meile et al. 2001). The RuMP cycle also regenerates Ru5P by way of X5P and Ru5P‐3‐epimerase, suggesting that flux to Ru5P in *B. methanolicus* will be affected by PktB activity on X5P. If X5P flux is diverted away from Ru5P via *pktB*, methanol assimilation rates could be decreased. Indeed, the methanol assimilation rate in the integrated *pktB* strain was slower when compared with the wild type (Figure 6b). In summary, while PKT expression improves biomass yield by reducing CO_2_ loss, the disruption of carefully balanced flux through the RuMP cycle could induce a fitness burden (Muller et al. 2015). All *pktB*‐expressing strains described in the present study experienced a growth defect, likely due to RuMP cycle imbalance.

In *B. methanolicus* , PktB activity increases the pool of AcP (Figure 5a), which can subsequently convert to AcCoA via phosphotransacetylase, or acetate and ATP via acetate kinase (Vadali et al. 2004). In contrast with a previous report of increased acetate excretion following *pktB* overexpression in its native host *M. buryatense* (Henard et al. 2017), the BMGA3::*pktB* strain excreted no detectable acetate (as compared with the wild type strain, which excreted ~3 mM). Although this observation suggests that *pktB* expression alters acetate metabolism in *B. methanolicus* , the underlying mechanism cannot be determined from the present data. One possibility is that the slower growth rate of the BMGA3::*pktB* strain reduces overflow metabolism and consequently decreases acetate excretion. Alternatively, carbon flux through acetyl‐phosphate may be preferentially directed toward acetyl‐CoA rather than acetate production. Distinguishing between these possibilities will require future metabolic flux analyses and/or intracellular metabolite measurements.

Although the present data do not distinguish the mechanism responsible for reduced acetate excretion, both proposed explanations predict increased availability of acetyl‐CoA. Previous fluxomic work in *B. methanolicus* indicates that under growth on methanol, the organism runs a partial non‐cyclic TCA cycle with a reductive branch that recycles pyruvate through oxaloacetate and malate, alongside an oxidative branch through acetyl‐CoA to citrate that operates with relatively low flux (Delepine et al. 2020). If *pktB* expression increases flux to acetyl‐CoA, the organism may compensate by driving more flux downstream to the oxidative TCA branch.

The contrasting phenotypes observed following PKT engineering across microbial hosts highlight an important principle of metabolic engineering: the physiological outcome of introducing a carbon‐conserving pathway is governed not only by the catalytic properties of the introduced enzyme, but also by the metabolic architecture into which it is integrated. Although PKT catalyses the same conserved reaction in each host, its impact on growth, carbon partitioning, and byproduct formation depends on the native organization of central carbon metabolism and the capacity of the cell to redistribute metabolic flux. Recent efforts to develop synthetic phosphoketolase‐based pathways similarly demonstrate that achieving the theoretical carbon‐conserving potential of PKT requires coordinated pathway engineering beyond introduction of the enzyme alone (Guo et al. 2023; Yang et al. 2023). Consistent with this concept, PKT expression in *B. methanolicus* increased biomass yield while reducing CO_2_ production without promoting acetate accumulation, a phenotype distinct from that previously reported in *M. buryatense* . These findings, along with recent synthetic biology studies, suggest that successful implementation of phosphoketolase‐mediated carbon conservation depends not only on introducing the pathway itself, but also on tailoring its integration to the metabolic architecture of the host organism.

To our knowledge, this is the first report of introducing a PKT into a native methylotroph lacking a PKT system. Organisms encoding both a PKT and the RuMP pathway enzymes in nature are predominantly Type I methanotrophs like *M. buryatense* and the closely related *Methylomicrobium alcaliphilum* 20Z^R^ (Akberdin et al. 2018; Kalyuzhnaya et al. 2013). The PktB in this work was taken from the native methylotroph *M. buryatense* , a Type I methanotroph that also assimilates C1 substrates via the RuMP cycle. While PKT effectively rewires carbon flux through non‐oxidative glycolysis in methylotrophic organisms, additional engineering of the central metabolic network in *B. methanolicus* would likely improve methanol assimilation rates and reduce CO_2_ loss. Given that native PKT‐ and RuMP‐harbouring hosts have likely evolved a finely tuned balance between pathway expression and substrate utilization, adaptive laboratory evolution of BMGA3::*pktB* presents an avenue to optimize flux through the PKT‐RuMP pathway and resolve the growth defect observed herein. In parallel, integration of xylose utilization genes (*xylABT*) into a PKT background represents a compelling strategy to increase Ru5P regeneration and further support flux through the PKT pathway (Li et al. 2025). Collectively, these findings provide insights into the metabolic impact of heterologous PKT expression in a methylotrophic host and position PKT as a broadly applicable strategy for carbon‐conserving metabolic engineering in methylotrophs, expanding the genetic and metabolic toolkit available for biomanufacturing from methanol.

## Author Contributions

**Jonathan R. Humphreys:** formal analysis, investigation, validation, visualization, writing – review and editing, writing – original draft. **Yael J. Toporek:** formal analysis, investigation, methodology, validation, visualization, writing – original draft, writing – review and editing. **Marcus S. Bray:** formal analysis, investigation, validation, visualization, writing – original draft. **Thomas J. Musselwhite:** formal analysis, investigation, validation. **Michael T. Guarnieri:** conceptualization, funding acquisition, methodology, project administration, resources, writing – review and editing, supervision. **Kimberly A. Rosenbach:** investigation. **Katie P. Michel:** investigation.

## Funding

This work was funded by the US Department of Energy, Office of Critical Minerals and Energy Innovation, Alternative Fuels and Feedstocks Office and US Department of Energy, Office of Science, Office of Biological and Environmental Research, Genomic Sciences Program SFA IMAGINE Biosecurity, under contract number DE‐AC36‐08GO28308; WBS 2.3.2.122.

## Conflicts of Interest

The authors declare no conflicts of interest.

## Supporting information


**Figure S1:** Growth profiles for pLM and pLM‐*sfGFP*. The experiments shown in panels A‐C were performed independently for different experimental objectives (aTc toxicity, *sfGFP* induction, and repeated inducer addition, respectively). (A) aTc was dosed between 0 and 1.2 μM at OD 0.2 in cultures harbouring the pLM plasmid. (B) Growth curve of pLM and pLM‐*sfGFP* strains with or without 0.8 uM aTc dosing (C) aTc was dosed in pLM‐*sfGFP* cultures at ODs 0.2, 0.4, and 0.8. All error bars show standard error of the mean (SEM) for 3 replicates.
**Figure S2:** Growth profiles of pLM and pLM‐*pktB* strains with aTc addition. Error bars show SEM for 3 replicates.
**Figure S3:** Growth profiles of constitutive strains. (A) Growth profiles for the constitutively expresseed *sfGFP*‐expressing strains. (B) Growth profiles for the constitutive promoter *pktB*‐expressing strains. All error bars show SEM for 3 replicates.
**Figure S4:** Relative *pktB* expression for the plasmid‐based and integrated *pktB*. Relative *pktB* expression in plasmid‐based *pdxK‐pktB* and integrated BMGA3::*pktB* strains is compared vs. housekeeping gene *rpoB* using RT‐PCR. All error bars display SEM of 3 replicates.
**Table S1:** Strains used in this study.
**Table S2:** Plasmids used in this study.
**Table S3:** Primers used in this study.
**Table S4.** Synthesized oligonucleotide sequences used in this study.

## Data Availability

The data that support the findings of this study are available from the corresponding author upon reasonable request.
