# Supplementary material for: Rewiring Carbon Metabolism in Bacillus methanolicus via Heterologous Phosphoketolase Expression Enhances Biomass Yield From Methanol and Reduces CO2 Loss
Source: Microb Biotechnol. 2026 Sep 1;19(9):e70430. doi: 10.1111/1751-7915.70430 (PMC13531358; doi:10.1111/1751-7915.70430)
Supplement: Supplementary file 1 [file MBT2-19-e70430-s001.docx]

**Supplementary Information for:**

***Rewiring carbon metabolism in Bacillus methanolicus via heterologous phosphoketolase expression enhances biomass yield from methanol and reduces CO₂ loss***

Yael Toporek^1ⴕ^, Jonathan R. Humphreys^1ⴕ^, Thomas J. Musselwhite^1^, Marcus S. Bray^1^, Katie Michel^1^, Kimberly A. Rosenbach^1^, Michael T. Guarnieri^1,2,3^*

^ⴕ^These authors contributed equally

1. Bioenergy & Sustainable Transportation Directorate, National Laboratory of the Rockies, Golden, CO 80401
2. Department of Chemical and Biological Engineering, Colorado School of Mines, Golden, CO 80401
3. Renewable & Sustainable Energy Institute, University of Colorado, Boulder, CO 80303

***Corresponding Author:**

Michael T. Guarnieri, Ph.D.

Email: [Michael.Guarnieri@nlr.gov](mailto:Michael.Guarnieri@nlr.gov)

15013 Denver West Pkwy

Golden, CO 80401

**S1A.**

**S1B.**

**S1C.**

**Supplementary Figure 1. Growth profiles for pLM and pLM-*sfGFP*.** The experiments shown in panels A-C were performed independently for different experimental objectives (aTc toxicity, *sfGFP* induction, and repeated inducer addition, respectively). (A) aTc was dosed between 0-1.2 uM at OD 0.2 in cultures harboring the pLM plasmid. (B) Growth curve of pLM and pLM-*sfGFP* strains with or without 0.8 uM aTc dosing (C) aTc was dosed in pLM-*sfGFP* cultures at ODs 0.2, 0.4, and 0.8. All error bars show standard error of the mean (SEM) for 3 replicates.

Supplementary Figure 2. **Growth profiles of pLM and pLM-*pktB* strains with aTc addition**. Error bars show SEM for 3 replicates.

**S3B.**

**S3A.**

Supplementary Figure 3. **Growth profiles of constitutive strains.** (A) Growth profiles for the constitutively expresseed *sfGFP*-expressing strains. (B) Growth profiles for the constitutive promoter *pktB*-expressing strains. All error bars show SEM for 3 replicates.

Supplementary Figure 4. **Relative *pktB* expression for the plasmid-based and integrated *pktB*.** Relative *pktB* expression in plasmid-based *pdxK-pktB* and integrated BMGA3::*pktB* strains is compared vs housekeeping gene *rpoB* using RT-PCR. All error bars display SEM of 3 replicates.

Supplementary Table 1. **Strains used in this study.**

| **Strain Name** | **Description** | **Source** |
| --- | --- | --- |
| wild type | wild type *B. methanolicus* MGA3 | ATCC 51375 ™ |
| YT1 | *B. methanolicus* MGA3 harboring pYT1 plasmid | This study |
| pLM | *B. methanolicus* MGA3 harboring pLM plasmid | This study |
| pLM-*sfGFP* | *B. methanolicus* MGA3 harboring pLM-*sfGFP* plasmid | This study |
| pLM-*pktB* | *B. methanolicus* MGA3 harboring pLM-*pktB* plasmid | This study |
| *dppE-sfGFP* | *B. methanolicus* MGA3 harboring pYT-*dppE-sfGFP* plasmid | This study |
| *icd-sfGFP* | *B. methanolicus* MGA3 harboring pYT- *icd-sfGFP* plasmid | This study |
| *pdxK-sfGFP* | *B. methanolicus* MGA3 harboring pYT- *pdxK-sfGFP* plasmid | This study |
| *tuf-sfGFP* | *B. methanolicus* MGA3 harboring pYT- *tuf-sfGFP* plasmid | This study |
| *dppE-pktB* | *B. methanolicus* MGA3 harboring pYT- *dppE-pktB* plasmid | This study |
| *icd-pktB* | *B. methanolicus* MGA3 harboring pYT- *icd-pktB* plasmid | This study |
| *pdxK-pktB* | *B. methanolicus* MGA3 harboring pYT- *pdxK-pktB* plasmid | This study |
| *tuf-pktB* | *B. methanolicus* MGA3 harboring pYT- *tuf-pktB* plasmid | This study |
| YT21 | *B. methanolicus* MGA3 harboring pYT21 plasmid | This study |
| YT24 | *B. methanolicus* MGA3 harboring pYT24 plasmid | This study |
| BMGA3::*pktB* | *B. methanolicus* MGA3 with *pktB* chromosomally integrated under *pdxK* promoter near purine cluster locus | This study |

Supplementary Table 2. **Plasmids used in this study**

| **Plasmid** | **Annotations** | **Reference** |
| --- | --- | --- |
| pHP13 | Cm^R^, Em^R^ *E. coli/Bacillus* spp. shuttle vector | Haima, Bron et al. (1987) |
| pUB110 | Km^R^ *Bacillus* spp. shuttle vector | Gryczan, Contente et al. (1978) |
| pUB110S | pUB110 derivative with *E. coli* ori BR322 added | Irla, Heggeset et al. (2016) |
| pYT1 | pUB110S with neomycin resistance gene replaced by *ahp* Kan^R^ gene | This study |
| pLM | pYT1 derivative, aTc inducible pxyl/tet locus and tetR gene under strong pmdh promoter | This study |
| pLM-*sfGFP* | pLM with *sfGFP* under *p_xyl/tet_* promoter | This study |
| pLM-*pktB* | pLM with *sfGFP* swapped for *Methylotuvimicrobium buryatense 5GB1C pktB* gene under *p_xyl/tet_* promoter | This study |
| pYT-*dppE-sfGFP* | pYT1 derivative, *sfGFP* under *Bacillus methanolicus* MGA3 *dppE* promoter, in *lac* cloning site | This study |
| pYT-*icd-sfGFP* | pYT1 derivative, *sfGFP* under *B. methanolicus icd* promoter, in *lac* cloning site | This study |
| pYT-*pdxK-sfGFP* | pYT1 derivative, *sfGFP* under *B. methanolicus pdxK* promoter, in *lac* cloning site | This study |
| pYT-*tuf-sfGFP* | pYT1 derivative, *sfGFP* under *B. methanolicus tuf* promoter, in *lac* cloning site | This study |
| pYT-*dppE-pktB* | pYT1 derivative, *M. buryatense* 5GB1C *pktB* under *B. methanolicus dppE* promoter, in *lac* cloning site | This study |
| pYT-*icd-pktB* | pYT1 derivative, *M. buryatense* 5GB1C *pktB* under *B. methanolicus icd* promoter, in *lac* cloning site | This study |
| pYT-*pdxK-pktB* | pYT1 derivative, *M. buryatense* 5GB1C *pktB* under *B. methanolicus pdxK* promoter, in *lac* cloning site | This study |
| pYT-*tuf-pktB* | pYT1 derivative, *M. buryatense* 5GB1C *pktB* under *B. methanolicus tuf* promoter, in *lac* cloning site | This study |
| pYT21 | pYT1 derivative, sfGFP under *B. methanolicus mdh* promoter between *repB* and *ori* | This study |
| pYT24 | pYT21 derivative, *M. buryatense* 5GB1C *pktB* under *B. methanolicus pdxK* promoter, with 3-kb homology arms for chromosomal integration upstream of purine cluster | This study |

Supplementary Table 3. **Primers used in this study.**

| **Primer** | **Sequence 5’ → 3’** | **Function** |
| --- | --- | --- |
| oMSB99 | TTTTTCTTGGAATTGTGCTGCTTAACGTGAGTTTTCGTTCCACT | Forward primer for amplification of BR322 ori from pHP13 for Gibson assembly into pUB110 to make pUB110S |
| oMSB100 | TGGTTTTGGTCGGCACTGCGCCATTCGCCATTCAGG | Reverse primer for amplification of BR322 ori from pHP13 for Gibson assembly into pUB110 to make pUB110S |
| Rot781 | CAGTGCCGACCAAAACCA | Forward primer for amplification of pUB110 for Gibson addition of BR322 ori from pHP13 to make pUB110S |
| Rot782 | CAGCACAATTCCAAGAAAAA | Reverse primer for amplification of pUB110 for Gibson addition of BR322 ori from pHP13 to make pUB110S |
| oLM46 | CAATTATTAGAGGTCATCGTTTAGAAAAACTCATCGAGC | Forward primer for amplification of *ahp* gene fragment for Gibson assembly into pUB110S to replace neomycin gene |
| oLM47 | CTGAAAAGGGAATGAGAATAATGAGCCATATTCAACGG | Reverse primer for amplification of *ahp* gene fragment for Gibson assembly into pUB110S to replace neomycin resistance gene |
| oLM44 | TATTCTCATTCCCTTTTCAG | Forward primer for amplification of pUB100S, removing neomycin resistance gene |
| oLM45 | ACGATGACCTCTAATAATTG | Reverse primer for amplification of pUB100S, removing neomycin resistance gene |
| oLM91 | AAGCTTGACCTGTGAAGTG | Forward primer for amplification of pUB110S for Gibson addition of p*_xyl/tet_-sfGFP* fragment to make pLMtetON3 |
| oCAH026 | CAGCTCACTCAAAGGCGG | Reverse primer for amplification of pUB110S for Gibson addition of p*_xyl/tet_-sfGFP* fragment to make pLMtetON3 |
| oMSB64 | ATTAAATACTGCCCTTCCACC | Forward primer for amplification of 239 bp upstream of the methanol dehydrogenase gene native to *B. methanolicus*; putative *mdh* promoter |
| oMSB65 | TCTTACTACCTCCTATTTATGTAAT | Reverse primer for amplification of 239 bp upstream of the methanol dehydrogenase gene native to *B. methanolicus*; putative *mdh* promoter |
| oLM58 | TATGGTTTTGGTCGGCACTGGTTAAGGGATTTTGGTCATGAGATT | Forward primer for amplification of p*_mdh-tetR_* fragment for Gibson assembly into pUB110S to make pLMtetON3 |
| oLM75 | CAAACTGGAACAACACTCAATGCCTTAGGATCTAGTCG | Reverse primer for amplification of f p*_mdh-tetR_* fragment for Gibson assembly into pUB110S to make pLMtetON3 |
| oLM71 | TTGAGTGTTGTTCCAGTTTGG | Forward primer for amplification of pUB110S for Gibson addition of p*_mdh-tetR_* fragment to make pLMtetON3 |
| oLM97 | GGGAAAAGGTGGTGAACTAC | Forward primer for amplification of *pktB* gene fragment for assembly into pLMtetON3 |
| oLM98 | TCACTTCACAGGTCAAGC | Reverse primer for amplification of *pktB* gene fragment for assembly into pLMtetON3 |
| oLM91 | AAGCTTGACCTGTGAAGTG | Forward primer for amplification of pLMtetON3-*sfGFP* backbone while removing *sfGFP* for Gibson assembly with *pktB* gene fragment |
| oLM92 | AGTAGTTCACCACCTTTTCCC | Reverse primer for amplification of pLMtetON3-sfGFP backbone while removing *sfGFP* for Gibson assembly with *pktB* gene fragment |
| YT179 | CAGTATTTAATTTGACTTTAAAAAGGATTAAAGTCATATCATA | Reverse primer for amplification of pYT backbone for Gibson addition with all Twist promoters and genes |
| YT199 | GCAACCAATTTTAAGGGTTTTCAATACTTTAAAACACATACATACCAACACCCTTCCACCTTAACCCTCCACACTATATAAGC | Forward primer for amplification of *sfGFP* to add to pYT1 backbone via Gibson assembly for later chromosomal integration of *pktB* |
| YT200 | GAAATAACGTCATTTTTATTTTAGTTGCTGAAAGGTGCGTTGAAGTTATTTGTAGAGCTCATCCATGCCATGTGTAATCC | Reverse primer for amplification of *sfGFP* to add to pYT1 backbone via Gibson assembly for later chromosomal integration of *pktB* |
| YT201 | CTTCAACGCACCTTTCAGCAACTAAAATAAAAATGACGTTATTTCTATATGTATCA | Forward primer for amplification of pYT1 backbone for adding *sfGFP* via Gibson assembly for later chromosomal integration of *pktB* |
| YT202 | TGTTGGTATGTATGTGTTTTAAAGTATTGAAAACCCTTAAAATTGGTTGCA | Reverse primer for amplification of pYT1 backbone for adding *sfGFP* via Gibson assembly for later chromosomal integration of *pktB* |
| oMSB73 | CAGTGCCGACCAAAACCA | Forward primer for amplification of pYT1 backbone for adding *sfGFP* via Gibson assembly for later chromosomal integration of *pktB* |
| oCAH181 | CCAGTCGGGAAACCTGTC | Reverse primer for amplification of pYT1 backbone for adding *sfGFP* via Gibson assembly for later chromosomal integration of *pktB* |
| YT230 | TGCAGCTGGCACGACAGGTTTCCCGACTGGGGGAGGACCATCTCCCAAGG | Forward primer for amplication of upstream 3-kb homology region of genomic DNA for construction of pYT31 via Gibson assembly; later chromosomal integration of *pktB* |
| YT204 | GCCTTTTGCAATCCTGACTTTTCGAAAGAAAAAAATGG | Reverse primer for amplication of upstream 3-kb homology region of genomic DNA for construction of pYT31 via Gibson assembly; later chromosomal integration of *pktB* |
| YT212 | GCCATTTTTTTCTTTCGAAAAGTCAGGATTGCAAAAGGCTTTTATCACCTCTTTATTAGCAGCCGTTTTCAGTGG | Forward primer for amplification of *pdxK-pktB* for construction of pYT31 via Gibson assembly; later chromosomal integration of *pktB* |
| YT210 | GGCGATCATTTGAAACGAGCCAGAATATCCGGATATAGTTCCTCCTTTCAGCAAAAAACC | Reverse primer for amplification of *pdxK-pktB* for construction of pYT31 via Gibson assembly; later chromosomal integration of *pktB* |
| YT205 | ATTCTGGCTCGTTTCAAATGATCGCCC | Forward primer for amplification of downstream 3-kb homology region of genomic DNA for construction of pYT31 via Gibson assembly; later chromosomal integration of *pktB* |
| YT231 | TTAAAGGTTTTATGGTTTTGGTCGGCACTGTGCTTGCTTGAAACGCTCAAGATTTCTTTT | Reverse primer for amplification of downstream 3-kb homology region of genomic DNA for construction of pYT31 via Gibson assembly; later chromosomal integration of *pktB* |
| YT235 | GGTTAAGTTAGAAAGGGCGC | Forward primer for confirmation of chromosomal integration at purine locus |
| YT234 | ACGGCAAATTTTTCAATTGAGGTC | Reverse primer for confirmation of chromosomal integration at purine locus |
| oKAR40 | TCGGATCACACGTAACACCG | Forward primer for qPCR; *pktB* locus |
| oKAR41 | ATTAGAGTGCCAGGCGGTTG | Reverse primer for qPCR; *pktB* locus |
| oKAR44 | TTCGTCCGGTTATCGCTTCC | Forward primer for qPCR; *rpoB* locus |
| oKAR45 | ACGGCCATAGTGGGAGTAGT | Reverse primer for qPCR; *rpoB* locus |

Supplementary Table 4. **Synthesized oligonucleotide sequences used in this study**

| **Oligo names** | **Sequence 5’ → 3’** |
| --- | --- |
| p*_xyl/tet_* | AGTCCTGTCGGGTTTCGCCACCTCTGACTTGAGCGTCGATTTTTGTGATGCTCGTCAGGGGGGCGGAGCCTATGGAAAAACGCCAGCAACGCGGCCTTTTTACGGTTCCTGGCCTTTTGCTGGCCTTTTGCTCACATGTTCTTTCCTGCGTTATCCCCTGATTCTGTGGATAACCGTATTACCGCCTTTGAGTGAGCTGGAATTCCAAAAACTAAAAAAAATATTGACACTCTATCATTGATAGAGTATAATTAACGGGATCCCCAGCTTGTTGATACACTAATGCTTTTATATAGGGAAAAGGTGGTGAACTACTAAGCTTGACCTGTGAAGTGAAAAATGGCGCACATTGTGCGACATTTTTTTTGTCTGCCGTTTACCGCTACTGCGTCACGGATCTCCACGCGCCCTGTA |
| p*_icd-gfp_* | TTATGATATGACTTTAATCCTTTTTAAAGTCAAATTAAATACTGAAGATTTTTTAATGCCAAAAAATAAAATCTCCATTTTACTTTTTTGAGAAAAATTGATGTAATAAAAGAGTGAGGAAAAAAGGTTAGGGGCAAAGCCTTCTAACCTTTGATTCTGATTTTTTACCAGCATTGCCCCTGGCTTTCTAAAAAGCTGCATTCGGCGGAGCTGATCCGCCGGCTTTTAGGAATGCTTTTTAAAAAGAAAAAAATAAATAGGAATGTTAGGGGCACTTAGCTCTTATAGAAGGGAGAAAAAATGAGCAAAGGAGAAGAACTT |
| p*_dppE-gfp_* | GATCTAGTCGATCAGTCGTTTTATGATATGACTTTAATCCTTTTTAAAGTCAAATTAAATACTGATTTGCAATGGAAGTGTGCGACAAAGTTTATCCTTTTCGTACATTTTTAAGCCAGGAGCACCATGTTGATTGCTGGCTCCAAGATGAAAGGGCGCAAAAGCTTTTTTTAACAAAAACATAAAAAGGTATCTGCCGGCTGCATCTTATACCAGCCGGTTTATATAGATACAAGAATTTTTTGAAAAAGAGGGGGAAAAGAGATGAGCAAAGGAGAAGAACTTTTCACTGGAGTTGTCCCAATTCTTGTTGAATTA |
| p*_pdxK-gfp_* | TTAGGATCTAGTCGATCAGTCGTTTTATGATATGACTTTAATCCTTTTTAAAGTCAAATTAAATACTGTTTTATCACCTCTTTATTAGCAGCCGTTTTCAGTGGTAAAGGGGTGGAGCTGTTGTTTTTAAATGAAGCCTATTTCCATATTTAGGGTAGGAGTTTAAAACTCATGCACAATTTCTGATGATTTTGCATACTATCATAAAATTTCGGTTTTTTCATTCAATAACCTTCATTTCGCGTTACTTATTCCAAAGCCCTTTCGTTTCTAAAATTTTGTACTCTCTTTTTGTAGTGATATAATTTAAAAATAAACCAATATTTGGACAATTAGACTACGAGGAGGAAGCATCTAATGAGCAAAGGAGAAGAACTTTTCACTGGAGTTGTCCCAATTCTT |
| p*_tuf-gfp_* | GGATCTAGTCGATCAGTCGTTTTATGATATGACTTTAATCCTTTTTAAAGTCAAATTAAATACTGTTTTGACCACTACGAAGAAGTACCGAAATCAATCGCTGATGAAATCATCAAAAAAAATAGAGGCGAATAATTGATTTTAGTCTTTTAATCAAGTATAACTACTTATGTAAGCTTGGTTGCTGCGAATTAGGGCAGCCTCTTTCGCAGCCCATTTATATATTTAAAATTAAATTTTTTATAACCAAAGGAGGATTTTCCTAATGAGCAAAGGAGAAGAACTTTTCACTGGAGTTGTCCCAATTCTTGTTGAATTAGAT |
| p*_icd-gfp_* | TTATGATATGACTTTAATCCTTTTTAAAGTCAAATTAAATACTGAAGATTTTTTAATGCCAAAAAATAAAATCTCCATTTTACTTTTTTGAGAAAAATTGATGTAATAAAAGAGTGAGGAAAAAAGGTTAGGGGCAAAGCCTTCTAACCTTTGATTCTGATTTTTTACCAGCATTGCCCCTGGCTTTCTAAAAAGCTGCATTCGGCGGAGCTGATCCGCCGGCTTTTAGGAATGCTTTTTAAAAAGAAAAAAATAAATAGGAATGTTAGGGGCACTTAGCTCTTATAGAAGGGAGAAAAAATGAGCAAAGGAGAAGAACTT |
| p*_dppE-gfp_* | GATCTAGTCGATCAGTCGTTTTATGATATGACTTTAATCCTTTTTAAAGTCAAATTAAATACTGATTTGCAATGGAAGTGTGCGACAAAGTTTATCCTTTTCGTACATTTTTAAGCCAGGAGCACCATGTTGATTGCTGGCTCCAAGATGAAAGGGCGCAAAAGCTTTTTTTAACAAAAACATAAAAAGGTATCTGCCGGCTGCATCTTATACCAGCCGGTTTATATAGATACAAGAATTTTTTGAAAAAGAGGGGGAAAAGAGATGAGCAAAGGAGAAGAACTTTTCACTGGAGTTGTCCCAATTCTTGTTGAATTA |
| p*_pdxK-gfp_* | TTAGGATCTAGTCGATCAGTCGTTTTATGATATGACTTTAATCCTTTTTAAAGTCAAATTAAATACTGTTTTATCACCTCTTTATTAGCAGCCGTTTTCAGTGGTAAAGGGGTGGAGCTGTTGTTTTTAAATGAAGCCTATTTCCATATTTAGGGTAGGAGTTTAAAACTCATGCACAATTTCTGATGATTTTGCATACTATCATAAAATTTCGGTTTTTTCATTCAATAACCTTCATTTCGCGTTACTTATTCCAAAGCCCTTTCGTTTCTAAAATTTTGTACTCTCTTTTTGTAGTGATATAATTTAAAAATAAACCAATATTTGGACAATTAGACTACGAGGAGGAAGCATCTAATGAGCAAAGGAGAAGAACTTTTCACTGGAGTTGTCCCAATTCTT |
| p*_tuf-gfp_* | GGATCTAGTCGATCAGTCGTTTTATGATATGACTTTAATCCTTTTTAAAGTCAAATTAAATACTGTTTTGACCACTACGAAGAAGTACCGAAATCAATCGCTGATGAAATCATCAAAAAAAATAGAGGCGAATAATTGATTTTAGTCTTTTAATCAAGTATAACTACTTATGTAAGCTTGGTTGCTGCGAATTAGGGCAGCCTCTTTCGCAGCCCATTTATATATTTAAAATTAAATTTTTTATAACCAAAGGAGGATTTTCCTAATGAGCAAAGGAGAAGAACTTTTCACTGGAGTTGTCCCAATTCTTGTTGAATTAGAT |
